# Green Synthesis, Characterization, and Antibacterial Properties of Silver Nanoparticles Obtained by Using Diverse Varieties of *Cannabis sativa* Leaf Extracts

**DOI:** 10.3390/molecules26134041

**Published:** 2021-07-01

**Authors:** Adriana Cecilia Csakvari, Cristian Moisa, Dana G. Radu, Leonard M. Olariu, Andreea I. Lupitu, Anca Ofelia Panda, Georgeta Pop, Dorina Chambre, Vlad Socoliuc, Lucian Copolovici, Dana Maria Copolovici

**Affiliations:** 1 Biomedical Sciences Doctoral School, University of Oradea, 1 University St., 410087 Oradea, Romania; alex2adi4zina@yahoo.com; 2 Faculty of Food Engineering, Tourism and Environmental Protection, Institute for Research, Development and Innovation in Technical and Natural Sciences, Aurel Vlaicu University, 2 Elena Dragoi St., 310330 Arad, Romania; moisa.cristian@yahoo.com (C.M.); poianarusca@yahoo.com (D.G.R.); leo_olariu@yahoo.com (L.M.O.); pag.andreea@yahoo.com (A.I.L.); dorinachambree@yahoo.com (D.C.); lucian.copolovici@uav.ro (L.C.); 3 Faculty of Agriculture, Banat University of Agricultural Sciences and Veterinary Medicine, King Michael 1st of Romania from Timisoara, 119 Calea Aradului St., 300645 Timisoara, Romania; anpaiu@yahoo.com (A.O.P.); getapop_tm@yahoo.com (G.P.); 4 Center for Fundamental and Advanced Technical Research, Romanian Academy–Timisoara Branch, Laboratory of Magnetic Fluids, Mihai Viteazul Ave. 24, 300223 Timisoara, Romania; vsocoliuc@gmail.com

**Keywords:** antioxidant activity, *Cannabis sativa*, green synthesis, silver nanoparticles, antibacterial activity, SEM-EDX, UHPLC-DAD-MS

## Abstract

*Cannabis sativa* L. (hemp) is a plant used in the textile industry and green building material industry, as well as for the phytoremediation of soil, medical treatments, and supplementary food products. The synergistic effect of terpenes, flavonoids, and cannabinoids in hemp extracts may mediate the biogenic synthesis of metal nanoparticles. In this study, the chemical composition of aqueous leaf extracts of three varieties of Romanian hemp (two monoecious, and one dioecious) have been determined by Fourier-Transformed Infrared spectroscopy (FT-IR), high-performance liquid chromatography, and mass spectrometry (UHPLC-DAD-MS). Then, their capability to mediate the green synthesis of silver nanoparticles (AgNPs) and their pottential antibacterial applications were evaluated. The average antioxidant capacity of the extracts had 18.4 ± 3.9% inhibition determined by 2,2-diphenyl-1-picrylhydrazyl (DPPH^•^) and 78.2 ± 4.1% determined by 2,2′-azino-bis (3-ethylbenzothiazoline-6-sulfonic acid) diammonium salt (ABTS™) assays. The total polyphenolic content of the extracts was 1642 ± 32 mg gallic acid equivalent (GAE) L^−1^. After this, these extracts were reacted with an aqueous solution of AgNO_3_ resulting in AgNPs, which were characterized by UV−VIS spectroscopy, FT-IR, scanning electron microscopy (SEM-EDX), and dynamic light scattering (DLS). The results demonstrated obtaining spherical, stable AgNPs with a diameter of less than 69 nm and an absorbance peak at 435 nm. The mixture of extracts and AgNPs showed a superior antioxidant capacity of 2.3 ± 0.4% inhibition determined by the DPPH^•^ assay, 88.5 ± 0.9% inhibition as determined by the ABTS^•+^ assay, and a good antibacterial activity against several human pathogens: *Escherichia coli, Klebsiella pneumoniae, Pseudomonas fluorescens,* and *Staphylococcus aureus.*

## 1. Introduction

From the three *Cannabis* subspecies, namely, *Cannabis sativa*, *Cannabis indica,* and *Cannabis ruderalis*, the most extensive crop is represented in Europe by *C. sativa*, with its varieties being harvested for their seeds, stalks, or biomass. The wide variety of applications of *Cannabis sativa* L. (hemp) have been known for centuries for their use in textile [1], paper, food [2,3,4], construction [5], medicine [6], etc., because of their phytochemical and fiber contents. A recent review depicted the applications of *C. sativa* from ancient times to the present, including the ethnological, botanical, chemical, and pharmacological features, with a focus on pharmaceutical research, in order to show the potential therapeutic activities of the plant [2]. *C. sativa* is a dioecious or monoecious annual plant of the family *Cannabinaceae,* which can reach up to 5 m. Nowadays, the vast publications on the chemical composition of diverse extracts of hempseeds, roots, and aerial parts (leaves, stems, and inflorescences) of the plant indicated the multitude of metabolites present in the extracts, depending on the hemp variety, meteorological and climatic conditions, and extraction methodology used, namely: solvents, temperature, time of extraction, method (maceration, classic extraction, ultrasound-assisted extraction, and microwave-assisted or supercritical fluid extraction), pH, pressure, etc. [7]. The main hemp phytochemicals that are synthesized in trichomes that cover the leaves, branches, and stems of the plant belong to the following classes: cannabinoids (more than 100), polyphenols, flavonoids, terpenoids (which give the smell of the crop), amides, amines, carbohydrates, fatty acids, and esters, with many of them having medical uses [8], alone or in diverse preparations. The results of several studies have demonstrated the involvement of phytocannabinoids in several central and peripheral pathophysiological mechanisms, namely: food intake (modulate hunger/satiety), multiple sclerosis, inflammation, pain [9], spasticity, colitis, nausea and vomiting, anorexia, Tourette syndrome, sleep disorders, anxiety, epilepsy, Alzheimer’s disease, and Parkinson’s disease [2,10,11]. Therefore, a strict pathway to obtain authorization for a potential medicinal product containing hemp preparations, which have little or no psychoactive effect because they contain low levels of Δ^9^-tetrahydrocannabinol (Δ^9^-THC), is required, and has been mentioned in a recent report from the European Monitoring Centre for Drugs and Drug Addiction [12].

Metal and metal-based nanoparticles (NPs) of various sizes, shapes, and compositions may be obtained by chemical or physical methods for industrial and medicinal applications [13,14] with good yields and quantities. Still, the synthesis steps present a potentially toxic effect on the environment [15,16]. Green biosynthesis, as a biological method suitable to obtain NPs, is easy to perform, less expensive, environmentally friendly, and very vast in the existence and access of starting material, namely organism material, because of the enormous plant kingdom metabolites/organisms strains [17,18,19,20,21,22,23]. Green synthesis of metal-based NPs could use fungi, bacteria, yeast, algae, and plant extracts. The latter has the most abundant scientific reports, with diverse plants being used to obtain AgNPs (e.g., *Urtica dioica* [24], *Paederia foetida* [25], and *Vetiveria zizanioides* [18,26]), AuNPs [18], CuNPs, ZnONPs, etc. [27,28,29,30,31,32,33,34]. *Cannabis sativa* extracts were used to obtain AgNPs [35,36,37], AuNPs [26,38], and Ag-AuNPs [39], and these were evaluated for their biological activity (antimicrobial effect, antifungal activity, and biofilm inhibition).

Hemp plants are mainly used for seeds and the leaves are considered as plant wastes. The present study aimed to use those leaves (from varieties able to produce a higher yield of oil from seeds) in order to obtain extracts rich in biomolecules and for their potential to mediate the green synthesis of NPs. The second aim of the study was the characterization of all plant extracts and their mixtures: plant extract + AgNPs by FT-IR, UV−VIS, SEM-EDX, DLS, and to test them against several human pathogens: *Escherichia coli, Klebsiella pneumoniae, Pseudomonas fluorescens,* and *Staphylococcus aureus*.

## 2. Results

### 2.1. Obtaining and Analyses of the Extracts

Three varieties of *Cannabis sativa* powdered leaves, two monoecious (Diana and Denise) and one dioecious (Silvana, female and male, respectively), were used to obtain aqueous yellow extracts using an ultrasound-assisted method. The extracts were analyzed by high-performance liquid chromatography, UV−VIS spectroscopy, FT-IR spectroscopy, and mass spectrometry (UHPLC-DAD-MS). The semi-quantitative and quantitative identified compounds are presented in Table 1. Ten carboxylic acids (22.95% concentration, e.g., cannabigerolic acid (CBGA) and cannabidiolic acid (CBDA)) and seventeen decarboxylated constituents (33.97%), flavonoids (cannflavin A: 3.56% and C: 3.58%), and canniprene (2.01%) were identified. Other unknown compounds were also observed (33.93%).

The total phenolics content (TPC) was determined using Folin−Ciocalteu reagent and a spectrophotometric method. The values of the TPC were determined both for the leaf extracts and the solution that remained after the biosynthesis of AgNPs, and the data are presented in Table 2. The TPC varied from 1581 mg GAE L^−1^ (CsSm) to 1721 mg GAE L^−1^ (CsSf) for the aqueous extracts, and a high decrease was determined for the solution remaining after the synthesis of the AgNPs.

### 2.2. Antioxidant Activity

The antioxidant activity of the extracts was evaluated using ABTS^•+^ and DPPH^•^ assays (Table 3), and the results ranged from 66.29% (CsDi extract) to 84.32% (CsSm extract) for ABTS^•+^ inhibition, and 0.23% (CsDe extract) to 0.73% (CsSm extract) for ABTS^•+^ mmol TEAC L^−1^, while for DPPH^•^ inhibition varied from 11.82% (CsSm extract) to 29.92% (CsSf extract), and from 7.60% (CsSm extract) to 17.32% (CsSf extract) for DPPH^•^ mg GAE/L. After the biosynthesis of the AgNPs, we determined the antioxidant activity for the mixture of extract + AgNPs and for the purified AgNPs. As can be seen from Table 3, the values of inhibition for the extract + AgNPs varied from 86.06% (PDi) to 90.33% (PSf), while for purified AgNPs it varied from 73.10% (NPDi) to 89.53% (NPSf), as determined by the ABTS^•^^+^ assay. The inhibition for the extract + AgNPs varied from 1.56% (PDe) to 3.39% (PDi), while for purified AgNPs it varied from 5.60% (NPDi) to 6.67% (NPSm), as determined by the DPPH^•^ assay.

### 2.3. UV−VIS Spectroscopy Analysis

**Figure 1:**
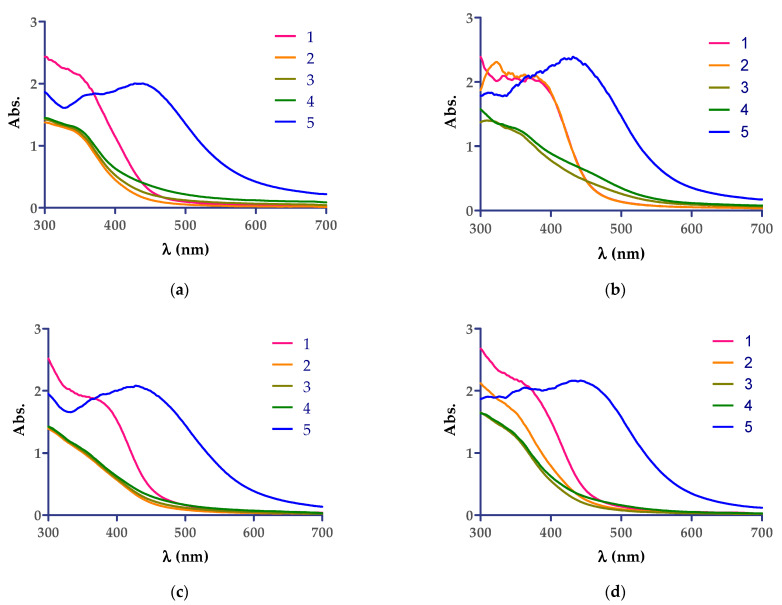
UV−VIS spectra showing the development of AgNPs by *Cannabis sativa* extracts: (**a**) CsDi, (**b**) CsDe, (**c**) CsSf, and (**d**) CsSm. The correspondence of the curves are as follows: 1 (red)—aqueous solution extract diluted 1:2 with distilled water; 2 (yellow)—mixture of extract + AgNO_3_ 5 mM after 1 min reaction time at room temperature; 3 (grey)—mixture of extract + AgNO_3_ 5 mM after 30 min reaction time at room temperature; 4 (green)—mixture of extract + AgNO_3_ 5 mM after 60 min of reaction time at room temperature; 5 (blue)—mixture of extract + AgNO_3_ 5 mM after 8 min reaction time at 90 °C.

The diluted aqueous *Cannabis sativa* leaf extracts were mixed with 5 mM AgNO_3_ aqueous solution and monitored visually and spectrophotometrically by scanning in the 300–700 nm range. The reaction was monitored at room temperature spectrophotometrically until 60 min from the mixing time of the solutions, as can be seen in Figure 1, and no AgNPs formation was observed. The color change from yellow to reddish-brown was observed in the case where the reaction mixture was kept at 90 °C for 8 min. The color change photos and maximum wavelength observed after thermal treatment of the mixture of AgNO_3_ aqueous solution with extracts by UV−VIS spectroscopy analyses are the following: CsDi extract: 439 nm, CsDe extract: 432 nm, CsSm extract: 436 nm, and CsSf: 444 nm, and indicating the formation of AgNPs.

### 2.4. FT-IR Spectroscopy

Figure S1a–d presents the FT-IR ATR spectra of the *Cannabis sativa* leaf extracts and that of the mixtures of extracts + biosynthesized silver nanoparticles (AgNPs). The spectra were recorded between 4000–600 cm^−1^, and the main observed bands are shown in Table 4.

In the 1500–1200 cm^−1^ range, an important change in the spectra of *Cannabis sativa* leaf extracts with biosynthesized AgNPs compared with *Cannabis sativa* leaf extract samples can be observed. For all AgNP samples, the band located at ~1405 cm^−1^ strongly diminished, and a new band appeared at ~1368 cm^−1^.

### 2.5. Characterization of AgNPs by SEM-EDX Analyses

**Figure 2:**
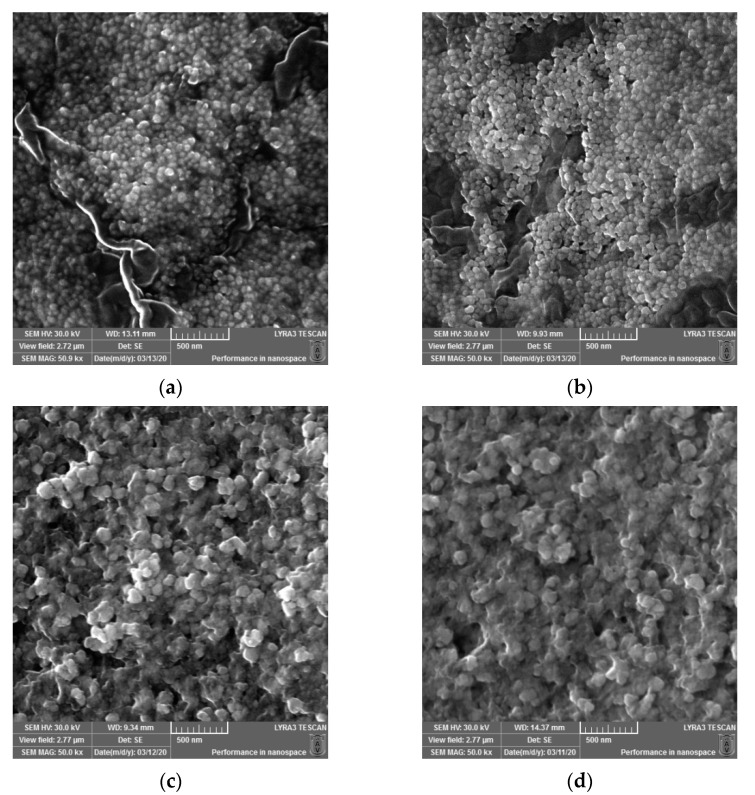
SEM micrographs recorded for the biosynthesized silver nanoparticles (AgNPs) obtained using *Cannabis sativa* leaves extracts: PDi (**a**), PDe (**b**), PSm (**c**), and PSf (**d**).

Micrographs were recorded using a scanning electron microscope (SEM) to determine the surface morphology and particle size of the newly synthesized AgNPs. The images at 50kx magnification were taken and are depicted in Figure 2. The sizes of the biosynthesized silver nanoparticles (AgNPs) obtained using *Cannabis sativa* leaves extracts were as follows: 48.07 ± 6.76 ^a^ for PDi (a), 48.93 ± 4.49 ^a^ for PDe (b), 65.45 ± 1.71 ^b^ for PSm (c), and 62.67 ± 8.52 ^b^ for PSf (d), where the superscript a and b denote significant differences between results after Tukey’s test for *p* < 0.05.

**Figure 3:**
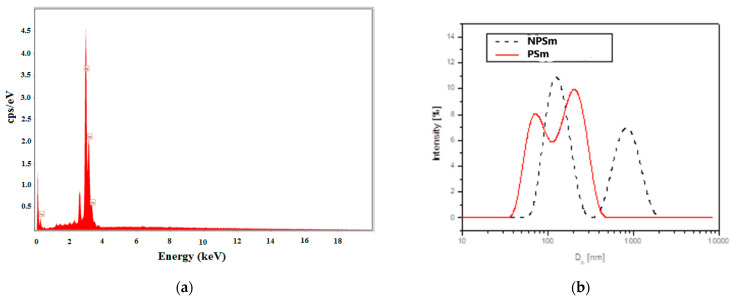
EDX spectrum for the mixture of AgNPs + CsSm extract (PSm) (**a**) and DLS hydrodinamic diameter distribution by intensity pattern (**b**) for the biosynthesized silver nanoparticles NPSm with a dotted line, and for the mixtureAgNPs + CsSm extract (PSM) with a red line.

The determination of the chemical composition of all AgNPs samples indicated by energy-dispersive X-ray (EDX) analyses revealed a strong peak at 3 keV, which is characteristic of AgNPs (Figure 3a) and confirms the specific regions of elemental silver (with 100% Ag on the spectrum). The mixtures of extracts and biosynthesized silver nanoparticles analyzed by EDX confirmed the presence of elemental silver in high quantities (more than 85%) and small quantities of oxygen and carbon from *Cannabis sativa* molecules.

### 2.6. Particle Size Distribution Analyses

From the DLS analysis, it was found that all of the samples showed nanoparticle aggregation. The size distribution by the intensity of the samples exhibited multimodal distributions with two or three principal modes. As an example, Figure 3b shows the size distribution (hydrodynamic diameter D_h_) by the intensity of CsSm and mixture of CsSm with AgNPs samples.

Table 5 summarizes the DLS data of the samples, a follows: Z-average hydrodynamic diameter (Z-Ave), the polydispersity index (PDI), and distribution peaks’ mode diameters and weights. One can notice that the Z-average diameter is greater than the SEM diameter for all of the samples, indicating nanoparticle clustering. From the comparison between the AgNPs and extracts + AgNPs data, it was found that the addition of the extract lowererd the aggregation degree in all of the samples, except the Denise samples.

### 2.7. Antibacterial Activity

The evaluation of the antibacterial activity of the *Cannabis sativa* leaf extracts without or with biosynthesized AgNPs was evaluated on four bacterial strains—three Gram-negative bacteria (*Escherichia coli*, *Klebsiella pneumoniae,* and *Pseudomonas fluorescens*), and one Gram-positive bacteria (*Staphyloccocus aureus*)—using a disc agar diffusion method (Figure S2).

Table 6 shows the antibacterial potency of the tested samples suing the reference antibiotics of Amikacin for *E. coli* and *K. pneumoniae*, Gentamicin for *P. fluorescens*, and *S. aureus*. Inhibition zones of about 7 mm were measured in plates with *E. coli*, *K. pneumoniae,* and *S. aureus,* seperately, and 0 mm for *Pseudomonas fluorescens* plates treated with *Cannabis sativa* leaf extracts, while for a mixture of leaf extracts and AgNPs, values between 10–12 mm were measured for *E. coli*, 10–13 mm for *P. fluorescens*, 12–13 mm for *S. aureus,* and 10–14 mm for *K. pneumonia*, respectively. The inhibition zones measured for the plates treated with antibiotics presented the following values: Amikacin: 20 mm for *E. coli* and 14 mm for *K. pneumoniae*, Gentamicin 20 mm for *P. fluorescens*, and 21 mm for *S. aureus.*

## 3. Discussion

The reduction of Ag^+^ ions into Ag^0^ nanoparticles was easily observed via the distinct color change from yellow-pale green to reddish and dark brown, respectively, due to the surface plasmon resonance phenomena. The best reaction conditions (the concentration of AgNO_3_ solution, the plant extract:AgNO_3_ solution ratio, the temperature, and the reaction time) for each plant extract that mediate the AgNPs synthesis should be investigated, and one recent article revealed these conditions for the following five plant extracts: *Berberis vulgaris* (root extract), *Brassica nigra* (seed extract), *Capsella bursa-pastoris* (leaves extract), *Lavandula angustifolia* (leaves extract), and *Origanum vulgare* (leaves extract) [40], and other articles presented the results related to *Cannabis sativa* extracts (hemp herd [37], leaf extracts [36,39], and hemp stem extracts [38]). To examine the formation of AgNPs UV−VIS spectroscopy experiments were conducted (Figure 1). The green synthesis of AgNPs was evaluated at different reaction times and conditions. The green synthesis performed at room temperature for 1 h did not lead to the preparation of AgNPs (Figure 1a–d). Still, it was rapid at 90 °C (about 8 min), a result that is in accordance with the earlier reported studies, where Sing et al. optimized the obtaining method of AgNPs from stem hemp extracts [38]. The best ratio of plant extract:AgNO_3_ 5 mM solution of 1:1 (*v*/*v*) was the most suitable, and the high temperature (90 °C) lead to the fast formation of AgNPs, a result that was confirmed by our study, and an increase in salt concentration lead to the appearance of a major shift in peak shift [38]. The formation of the AgNPs in suspension was confirmed by UV−VIS spectroscopy, as single, broad and strong surface plasmon resonance peaks, were observed in solutions containing plant extract, as follows: (i) CsDi: 439 nm, (ii) CsDe: 432 nm, (iii) CsSm extract: 436 nm, and (iv) CsSf: 444 nm, and indicated the formation of AgNPs. These results were in good agreement with the 350–500 nm peaks found for the plant extract mediated AgNP preparation suspensions reported [31,35,41].

The specific functional groups from the plant extracts and those bonded with the synthesized AgNPs were determined by FT-IR ATR spectroscopy in order to identify the possible biomolecules responsible for the reduction, capping, and efficient stabilization of AgNPs. In Figure 1a–d, the FT-IR ATR spectra of the *Cannabis sativa* leaf extracts and that of the mixture of extracts and AgNPs samples are depicted. The analysis of the obtained spectra suggests good similarities between the plant extracts samples and the corresponding mixtures with AgNPs. The bands recorded between 3550–3100 cm^−1^ are associated with –OH stretching vibration from alcohols and phenols structure [13,38,39,42], and with –NH stretching vibration of amines and amides groups [25,35,43], respectively, from proteins molecules [35,44]. In the 3100–2850 cm^−1^ range, the C–H and –CH_2_ stretching vibrations from the aliphatic structures [24,35,39,45] and =C–H (sp^2^ C) stretching from alkenes and aromatic structures [39] were recorded.

Between 1700–1500 cm^−1^, a large and strong band was observed. Similar behavior was reported by Singh et. al. [38] for the green synthesis of gold and silver nanoparticles from *Cannabis sativa* (industrial hemp) and this band was assigned to C=O carbonyl stretching from amides and to C=C stretching (in-ring) from aromatic structures. Some authors [4,43,46] mentioned that this wavenumber range may overlap the –NH bending (in-plane) vibration and C-N stretching vibration from protein structures. It should be noted that all samples containing AgNPs showed a lower intensity for the 1700–1500 cm^−1^ band than the corresponding leaves of the *Cannabis sativa* extracts. This behavior suggests that the protein molecules present in the plant extract are involved with functional groups (through the donated electrons pair to the surface of metal nanoparticles) in the reduction of Ag^+^ being an efficient capping agent in nanoparticle production and stabilization. The literature data report that proteins can bind to AgNPs through the free amine groups, and therefore, the stabilization of the nanoparticles by surface-bound proteins occurs [3,24,25,38,47].

The intense band located around 1405 cm^−1^ (CsDi: 1404.76 cm^−1^; CsDe: 1407.54 cm^−1^; CsSf extract: 1405.00 cm^−1^; CsSm extract: 1405.01 cm^−1^) was recorded and corresponded to –C–OH bending from alcohols or phenols groups [39]. In the AgNPs containing samples, the intensity at the ~1405 cm^−1^ band strongly diminished, and only a shoulder was recorded in the FT-IR spectra. At the same time, a new peak appeared at ~1368 cm^−1^, which, according to Aramwit et al. [48], can arise as a result of –COO^–^ stretching vibration of carboxylate groups. Abbasi et al. [39] considered that this band was due to the –C–O like phenol groups type. These results indicate that some phytochemicals (i.e., phenols, flavonoids, cannabinoids, polysaccharides, water-soluble biomolecules, etc.) with free hydroxyl groups from the *Cannabis sativa* extract are responsible for reducing Ag^+^ to Ag^0^, while other biomolecules (i.e., proteins) with –COOH or –OH functional groups rapidly bind the metal ions and are entrapped on the surface of the nanoparticles. Obviously, in this mode, the formation of mostly stable complexes between nanoparticles and effective plant extract functional groups leads to the stabilization of AgNPs, and prevents their aggregation. The obtained results are in good agreement with those reported by Singh et al. [38], which showed that AgNO_3_ and (–OH) reaction leads to Ag^0^ particles that undergo aggregation to form clusters. The formatted clusters further act as nucleation centers and catalyze the reduction of residual Ag^+^ ions.

It should be noted that the N-O stretching of the nitro compound gave the FT-IR signal in the 1550–1500 cm^−1^ and 1372–1290 cm^−1^ range, and could influence the recorded bands of the AgNPs. The shoulder recorded at ~1268 cm^−1^ in the plant extracts and the corresponding AgNPs containing mixtures could be attributed to the amide III band [48,49].

The peak recorded at ~1043 cm^−1^ could be attributed to C–N stretching vibration from amines or amides groups [38,39,43,47] and to –C–O–H bending vibration [1,39]. Therefore, the FT-IR spectra of synthesized NPs containing samples indicated the presence of proteins and hydroxyl compounds on the surface of synthesized AgNPs. The out of plane bending vibrations of =C–H (sp^2^ C) from the aliphatic structures of terpenes, flavonoids, and cannabinoids (i.e., cannabigerol, cannabigerolic acid, cannaflavin, β-myrcene, etc.) from *Cannabis sativa* extract and entrapped on AgNPs particles surface were recorded at ~923 cm^−1^, while the =C–H bending from the aromatic groups of protein appeared at ~780 cm^−1^ and 824 cm^−1^ [50]. According to Senthil et al. [42], the presence of the –OH and C=C moieties on the surface of the AgNPs is the signature of polyphenols, which are the essential phytochemicals constituents of plant extract.

The –C–N stretching vibration was recorded at ~870 cm^−1^ [39] and the N–H wagging [48] between 800–850 cm^−1^. The intensity of this last band increase for the AgNPs containing samples compared with plants extract samples because of the formation of amine salt (NH_2_^+^), which, according to Aramwit et al. [48], stabilized the AgNPs and prevented their precipitation or aggregation. The bands recorded between 750–600 cm^−1^ could be attributed to the stretching vibration of C–Cl and C–Br, which are characteristics of alkyl halides [38].

The FT-IR ATR results (Table 4) and the values of the total phenolic (Table 2) contents for the obtained *Cannabis sativa* leaves extracts and corresponding AgNPs containing solutions showed that the proteins and other biomolecules with -OH functional groups bind the metallic surface of nanoparticles and stabilized them by preventing their agglomeration. This is shown also in the SEM micrographs (Figure 3) and was confirmed by the DLS data (Table 5). The conformational changes that occurred in the protein molecules lead to more advanced exposure of the hydrophobic groups existing in their structure, relative to the aqueous solvent. This behavior can favor the actions of reducing agents (especially phenolic compounds) from plant extracts and the transformations of entrapped metal into metal nanoparticles, as it was demonstrated by decreasing the values of the total phenolic content by 6.9 times for CsDe to 8.11 times for CsSf (Table 2).

The tentatively qualitative analysis of the secondary metabolites present in *Cannabis sativa* leaf aqueous extracts was carried out using RP-UHPLC coupled with UV−VIS spectroscopy and mass spectrometry detectors, and the data were compared with that from the literature [50,51]. The presence of ten carboxylic acids (e.g., cannabigerolic acid (CBGA) and cannabidiolic acid (CBDA)) and seventeen decarboxylated constituents, flavonoids (cannflavin A and C), and canniprene was recognized. The data revealed a complex heterogeneous mixture of metabolites extracted in water, and many MS peaks were not assigned to any hemp components extracted in an aqueous mixture of solvents (e.g., methanol, ethanol, and acetone) or other polar solvents (e.g., alcohols and acetone) identified in other studies [52,53,54]. For the phenolic compounds, we identified pyrogallol, gallic acid, quercetin, and kaempferol.

The antioxidant capacity of the extracts and that of extracts + AgNPs were evaluated using the following two assays: DPPH^•^ and ABTS^•+^. There were no correlations between the results obtained, with the values of % inhibition as determined by the ABTS^•+^ assay being higher (average 78.2 ± 4.1%) compared with that obtained by the DPPH^•^ assay (average 18.4 ± 3.9%). The difference in these values arised from the difference in their principles of actions and the solubilisation of the radicals employed: the DPPH^•^ assay determined the ability of the tested samples that contain phenolic compounds and their derivatives to act as donors of hydrogen to the 2,2-diphenyl-1-picrylhydrazyl free radical, suitable in hydrophobic systems, while in the ABTS^•+^ assay, an electron-transfer methodthe, the ABTS^•+^ radical can react with more compounds, both in hydrophilic and hydrophobic systems, and lead to obtaining higher values than those calculated for the DPPH^•^ assay [55,56,57,58].

The surface morphology of the synthesized AgNPs was studied via SEM (Figure 3a–d). The particles were almost spherical and were well dispersed, with less than 65 nm for the AgNPs obtained from the leaf extract of dioecious Silvana variety, and less than 49 nm for the AgNPs formed by the leaf extracts monoecious varieties (Diana and Denise). The biomolecules involved in the Ag^+^ reduction and the stabilization of biogenic AgNPs conferred to the synthesized nanoparticles an eco-friendly nature. Singh et al. determined a similar spherical morphology and size (less than 25 nm) for the synthesized AgNPs using an aqueous extract of fresh leaves of *Cannabis sativa* harvested in India [35,38]. In comparison, Abassi et al. obtained AgNPs with less than 45 nm in diameter using *Cannabis sativa* leaf extract harvested in Pakistan [39]. Different parts of plant extracts mediated the production of AgNPs, with sizes that varied from 4 to 100 nm, as was reviewed for bark extracts by Burlacu et al. [59] or for plant leaf, fruit, and root extracts by El-Seedi et al. [60]. The Z-average diameter was greater in solution, as was determined by DLS, compared with the SEM diameter for all samples, which indicated nanoparticle clustering. From the comparison between the AgNPs and extracts + AgNPs data, it was found that the presence of both leaf extracts and AgNPs lowered the aggregation degree in all of the samples, except the CsDe samples.

The antibacterial effect of *Cannabis sativa* leaf extracts alone and its mixture containing prepared AgNPs was evaluated against both Gram-positive and Gram-negative bacterial strains using the disc diffusion method. Table 6 showed no significant antimicrobial activity for aqueous plant extracts against *Pseudomonas fluorescens,* and only a moderate effect against *E. coli*, *K. pneumonia*, and *S. aureus.* The synergistic antibacterial effect of mixtures of plant extracts + AgNPs was observed to be less (for *P. fluorescens, E. coli*, and *S. aureus,* giving 10–13 mm inhibition zones) compared with the antibacterial positive control (Amikacin: 20 mm for *E. coli*, and Gentamicin 20 mm for *P. fluorescens*, and 21 mm for *S. aureus*). For a mixture of leaf extracts and AgNPs, values between 10–14 mm inhibition zones for *K. pneumonia* were determined, with values very close to those obtained for the positive control (Amikacin: inhibition zone 14 mm). The present data obtained were in good agreement with that published in earlier reports: AgNPs got from green synthesis mediated by *Cannabis sativa* leaf extracts harvested in Pakistan have antibacterial effect against *K. pneumonia*, *B. subtilis*, *E. coli*, *S. aureus*, and *P. aeruginosa* [39]; the data obtained for the synergic effect of the mixture of *Cannabis sativa* leaf extracts harvested in India and antibiotics against *S. aureus, B. subtilis, E. coli, K. pneumonia,* and *S. marcescens* [35]; the AgNPs prepared using whole stems extracts of *Cannabis sativa* harvested in France were effective against biofilms of *E. coli* and *P. aeruginosa* [38]. The mechanism of action of AgNPs as antibacterial agents includes the damage of the bacterial cell membrane, the influence on the enzymatic system of the respiratory chain, and also DNA fragmentation, which have been earlier reported [61]. Earlier published data confirm that there is a higher inhibitory effect of AgNPs and Ag^+^ ions against the Gram-negative bacteria, because of the thicker peptidoglycan layer of Gram-positive bacteria, which can obstruct the action of Ag^+^ ions [62]. This data confirmed the potential activity of the mixture of *Cannabis sativa* leaf extract and AgNPs against different bacteria, with the best results shown for *Klebsiella pneumonia.*

## 4. Materials and Methods

All of the reagents and solvents used in the experiments were of adequate analytical grade and were obtained from Sigma-Aldrich and Merck (Merck KGaA, Darmstadt, Germany, and/or its affiliates).

### 4.1. Sample Collection and Preparation

Leaves from three varieties of Romanian hemp—two monoecious, Diana (named CsDi) and Denise (named CsDe), and one dioecious, Silvana (namely Silvana female, named CsSf, and Silvana male, named CsSm)—were harvested at the Agricultural Research and Development Station in Lovrin, Timis county, Romania, 45.548° N/20.461° E). The voucher specimens were kept at Banat University of Agricultural Sciences and Veterinary Medicine King Michael 1st of Romania from Timisoara. The leaves were deep-frozen until the experiments were performed. The leaves were dried in an oven (Binder, Tuttlingen, Germany) at 45 °C for three days and were then ground with a coffee mill (ZASS, model Zass ZCG 10, Hamburg, Germany) until a fine powder was obtained. A mixture of 1 g of sample and 10 mL of ultrapure water was kept in an ultrasonic bath at 30 °C for 30 min (Bandelin Sonorex, model Sonorex Super, Bandelin Electronic GmbH and Co. KG, Berlin, Germany). Then, the extracts were filtered through a 0.45 μm filter (Corning, Wiesbaden, Germany).

### 4.2. Determination of the Chemical Composition of the Extracts of Cannabis Sativa by UHPLC-DAD-MS

To analyze the chemical composition of the aqueous extracts of *Cannabis sativa* varieties, a high-performance liquid chromatograph coupled with a diode array detector and a mass spectrometer detector UHPLC-DAD-MS, (UHPLC: Nexera X2, DAD model M30A, MS Model 8040, Shimadzu, Tokyo, Japan) was employed. A Nucleosil HPLC C-18 reversed-phase column (EC 100/4.6 Nucleosil 100-3 C18, 100 × 4.6 mm^2^, particle size: 3.0 µm, length: 10 cm, Macherey-Nagel GmbH and Co. KG, Düren, Germany) was employed for the chromatographic separation. The mobile phase of the gradient elution of the method of separation of the compounds used was as follows: (A) distilled water with 0.1% trifluoroacetic acid and (B) acetonitrile with 0.1% trifluoroacetic acid. The gradient was applied at a flow rate of 0.5 mL/min, and the binary gradient with linear interpolation was used as follows: 0 min, 5% B; 5 min, 42% B; 25 min, 35%B; and 5 min, 5% B. The column and samples were thermostated at 25 °C. The injection volume of the sample was 10 μL. The mass spectra were recorded using a positive ionization mode (ESI + mode). The dry nitrogen was heated to 250 °C, and the drying gas flow was 15 L min^−1^. Data were acquired in the positive scan mode in the range 15–1500 Da. The compounds were identified by their UV–VIS spectra ranging from 190 to 600 nm, by comparing their retention times with the standards, and by analyzing their recorded mass spectra. To identify the cannabinoid compounds, we used the method reported by Brighenti et al., 2017 [63].

### 4.3. Total Phenolic Content

The total amount of phenolic compounds for the obtained extracts was determined using Folin−Ciocalteu (FC) reagent, as described earlier [64]. The total phenolics were determined and expressed as mg Gallic acid equivalents (GAE L^−1^) using a standard curve as a reference. Briefly, the leaf extracts were diluted with distilled water (1:25, *v*/*v*). In a volumetric flask, the following were added: 1 mL of sample, 0.5 mL FC reagent, 2 mL Na_2_CO_3_ (20%), and 5 mL distilled water. After 90 min of reaction at room temperature in the dark, the absorbance was recorded at λ = 765 nm using a UV−VIS spectrophotometer Specord 200, model Specord 200 Double-Beam (Analytik Jena GmbH, Jena, Germany), using a 10 mm quartz cuvette. The standard reference curve for gallic acid was recorded by measuring the absorbance of the solutions with concentrations of 0, 20, 40, 100, 160, and 200 mg L^−1^, respectively. The regression equation and correlation coefficient were calculated and then expressed in mg GAE L^−1^. All of the measurements were performed in triplicate.

### 4.4. Antioxidant Activity Assay

#### 4.4.1. 2,2-Diphenyl-1-Picrylhydrazyl (DPPH^•^) Assay

The antioxidant capacity of the extracts was evaluated using a spectrophotometric assay (2,2-Diphenyl-1-picrylhydrazyl: DPPH^•^ assay) as reported earlier. First, 0.1 mL of sample was mixed with 3 mL of 0.2 mM ethanolic DPPH^•^ solution. Ethanol was used as a negative control. After 60 min of reaction in the dark at room temperature, the absorbance was recorded using a UV−VIS spectrophotometer Specord 200, model Specord 200 Double-Beam (Analytik Jena GmbH, Germany) at λ = 517 nm and a 10 mm quartz cuvette. As a reference, positive controls containing 2.5–50 mg L^−1^ gallic acid were prepared. All of the experiments were conducted in triplicate, and the results were expressed as % inhibition (calculated as in [65]) and mg gallic acid equivalent L^−1^ (GAE L^−1^).

#### 4.4.2. 2,2′-Azino-bis(3-ethylbenzothiazoline-6-sulfonic acid) Diammonium Salt (ABTS^•+^) Assay

The antioxidant capacity of the extracts and AgNPs was determined using the scavenging activity of the ABTS^•+^ (2,2′-Azino-bis(3-ethylbenzothiazoline-6-sulfonic acid) diammonium salt) radical, following the method reported by Sridhar and Charles [66] with slight modifications. The ABTS™ solution reagent stock solution was prepared by mixing equal quantities of ABTS^•+^ reagent and 2.45 mM aqueous solution of sodium persulfate. The mixture was allowed to react for free radical generation at room temperature, overnight (12–16 h). To analyze the scavenging activity of the samples, 1 mL ABTS^•+^ solution was mixed with 0.5 mL of sample. The negative control was obtained in the same manner using ultrapure water. The absorbance was recorded after 10 min of incubation time in the dark, using a UV−VIS spectrophotometer Specord 200, model Specord 200 Double-Beam (Analytik Jena GmbH, Germany) at λ = 734 nm and a 10 mm quartz cuvette. For plotting the calibration curve, different (±)-6-Hydroxy-2,5,7,8-tetramethylchromane-2-carboxylic acid (TROLOX) standard concentrations were used (0.025–0.5 mM). All of the experiments were conducted in triplicate, and the results were expressed as % inhibition (calculated as in [66]) and mmol Trolox Equivalent Antioxidant Capacity L^−1^ (TEAC L^−1^) [66], respectively.

### 4.5. Biosynthesis of AgNPs

The aqueous extract was centrifugated at 8000 rpm for 3 min at the Hettich Rotina 380 R centrifuge. Then, 500 μL extract was mixed with 500 μL 5 mM AgNO_3_ aqueous solution and 700 μL purified water. This mixture was allowed to react for (a) 1 min at room temperature, (b) 30 min at room temperature, (c) 60 min at room temperature, and (d) 8 min at 90 °C. A similar optimization method was published in [38]. The synthesis procedure was repeated twice for each sample. The last method used (d) lead to the formation of AgNPs. Aliquots of this mixture were used to purify the AgNPs (named NPDi, NPDe, NPSm, and NPSf), which were washed and lyophilized, and some aliquots were further used as a mixture of AgNPs + extract (named PDi, PDe, PSm, and PSf).

### 4.6. UV−VIS Spectroscopy Analysis

The metal ions bio-reduction into metal nanoparticles was monitored by scanning in the 300–700 nm range, using a UV−VIS ScanDrop Nano-volume Spectrophotometer from AnalytikJena (Germany). The metal ion reduction was observed by a simple color change from pale green or yellow to a reddish-brown, presenting a high absorbance at 450 nm, revealing the formation of AgNPs. The quartz cuvette of a 1 cm light path length was used, and the light absorption spectra were given in reference to distilled water.

### 4.7. FT-IR Spectroscopy

The FT-IR ATR experiments were performed on lyophilized samples of the leaf extracts of *Cannabis sativa* varieties and the mixture of extracts with synthesized AgNPs containing solutions, respectively. The spectra were acquired over the range of 4000–600 cm^−1^ at a resolution of 4 cm^−1^ and 36 scans using a Bruker Vertex 70 spectrophotometer equipped with the ATR cell. Before each measurement, the background calibration was done. OPUS software was used to acquire and process the experimental data (spectra normalization and baseline correction). The intensity of absorption bands was normalized against the absorption at around 1043 cm^−1^ (min/max).

### 4.8. Characterization of AgNPs by SEM-EDX Analyses

A scanning electron microscope (SEM; LYRA 3 XMU, Tescan, Czech Republic) operated at 30 kV was used to determine the morphology and particle size of the AgNPs. Several droplets of the solution were put on the stub covered with carbon tape, and images at 50kx magnification were taken. Elemental analysis was performed using Energy Dispersive X-ray (EDX) spectroscopy (EDAX Inc., Mahwah, NJ, USA).

### 4.9. Particle Size Distribution Analyses

The colloidal stability of the samples was investigated using dynamic light scattering (DLS) using a Malvern Nano Zetasizer ZS instrument (Malvern Panalytical Ltd., Malvern, UK). The measurements were done at 25 °C in a backscattering configuration (173°).

### 4.10. Antibacterial Activity

Antibacterial efficiency against four bacterial strains—three Gram-negative bacteria (*Escherichia coli* (ATCC 25922), *Klebsiella pneumonia* (ATCC 13883), and *Pseudomonas fluorescens* (ATCC 27853)) and one Gram-positive bacteria *Staphyloccocus aureus* (ATCC 25923; Bio-Rad Laboratories (Hercules, CA, USA)) was analyzed using a disc agar diffusion method. Microbiological media (tryptic soy agar (TSA), tryptic soy broth, and Müeller−Hinton agar) were purchased from Roth GMBH Co KG (Nürnberg, Germany). Antimicrobial susceptibility test discs and the discs dispenser (Gentamicin (10 µg/disc), Amikacin (30 µg/disc)) were purchased from Oxoid Ltd. (Hampshire, U.K.). McFarland Standards for turbidity were purchased from GrantBio Ltd. (Chelmsford, Essex, U.K.). Inocula of bacterial cells (cultivated 24h at 35 °C on TSA) were suspended in sterile physiological saline to a density of 0.5% McFarland standard (equivalent to a concentration of 2.0 × 10^8^ cfu mL^−1^), then 1 mL bacterial suspension was applied to Petri dishes (Φ = 10 cm) containing Müeller−Hinton agar. After 5 min, the excess fluid was removed, and the inoculated Petri dishes were left to dry at room temperature for approximately 10 min. The predetermined battery of 6 mm in diameter of discs for each plate included four types of impregnated discs: (i) a reference antimicrobial disc (Amikacin 30 μg for *E. coli* and *K. pneumoniae*, and Gentamicin 10 μg for *P. fluorescens* and *S. aureus*), (ii) 50 μL of crude extract (CsDi, CsDe, CsSm, and CsSf), (iii) 50 μL mixture of plant extract + silver nanoparticles (PDi, PDe, PSm, and PSf), and (iv) 50 μL ultrapure sterile water (negative control), respectively. Discs were aseptically applied to the surface of each of the inoculated plates using a disc dispenser so as to ensure complete contact with the agar surface and even distribution so that they were no closer than 24 mm from the center to center. The disk agar diffusion method was performed in triplicate for each extracted sample. Inoculated plates were incubated for 24 h at 35 °C. Inhibition zones were measured with sliding calipers and were expressed in mm as the diameters of clear zones around te discs. The results were expressed as the mean value of three independent analyses.

#### 4.11. Statistical Analysis

All of the statistical analyses were conducted with GraphPad Prism (version 5.0 for Windows, GraphPad Software, San Diego, CA, USA), and the statistical Tukey’s Multiple Comparison Test was performed. Data among species were compared with ANOVA followed by Tukey’s post hoc test, and means with different letters were significantly different at *p* < 0.05.

## 5. Conclusions

The preparation of silver nanoparticles (AgNPs) was successfully performed using aqueous extracts of different varieties (two monoecious and one dioecious) of leaves from *Cannabis sativa* with both reducing and stabilizing properties. The biomolecules present in the leaf extracts, determined by UHPLC-UV-MS and FTIR ATR analyses, mediated a rapid, simple, and eco-friendly reaction that reduced Ag^+^ to Ag^0^ as spherical AgNPs with less than 69 nm, as was revealed by the SEM analysis. The synergic activity of a mixture of AgNPs and leaf extracts was effective against all Gram-positive and Gram-negative bacteria tested. Further studies are needed to develop suitable technology based on *Cannabis sativa* extracts in order to obtain nanoparticles with specific size and morphology to be used in medicinal and biotechnological applications.

## Figures and Tables

**Table 1:** The chemical composition of the aqueous extracts determined by UHPLC-DAD-MS.

Compound	CsDi	CsDe	CsSf	CsSm
**Phenollic Compounds**				
Pyrogallol (mg L^−1^)	0.44 ± 0.03	0.39 ± 0.03	0.67 ± 0.02	0.35 ± 0.04
Gallic acid (mg L^−1^)	118.91 ± 0.71	95.46 ± 5.42	137.30 ± 9.85	96.14 ± 4.79
Quercetin (mg L^−1^)	28.60 ± 0.39	35.14 ± 0.09	28.15 ± 0.15	24.26 ± 0.20
Kaempferol (mg L^−1^)	2.07 ± 0.06	5.93 ± 0.14	0.39 ± 0.01	1.71 ± 0.03
**Cannabinoid Acids**				
Cannabidioloc acid (CBDA) (%)	0	0.037 ± 0.001	0.006 ± 0.008	0.195 ± 0.009
cannabigerolic acid (CBGA) (%)	0.086 ± 0.019	0.104 ± 0.004	0.945 ± 0.006	0.052 ± 0.015

**Table 2:** The total phenolics content (TPC) determined for the aqueous extracts and the remaining solution after the biosynthesis of the AgNPs was performed. The analyses were performed in triplicate (*n* = 3).

TPC (mg GAE L^−1^)	CsDi	CsDe	CsSf	CsSm
TPC1: Aqueous extract	1601.09 ± 0.5 ^a^	1663.97 ± 0.17 ^b^	1721.79 ± 0.26 ^c^	1581.86 ± 0.34 ^d^
TPC2: Solution remained after the biosynthesis of AgNPs	212.44 ± 0.28 ^a^	241.54 ± 0.48 ^b^	212.82 ± 0.36 ^a^	213.91 ± 0.32 ^c^
TPC1: TPC2	7.55	6.9	8.11	7.42

The results are presented as mean ± standard deviation and superscript different letters (^a–d^) denote significant differences between results on the same line of data after Tukey’s test for *p* < 0.05. Means with superscripts having the same letter in the line are not significantly different.

**Table 3:** The antioxidant activity was determined by ABTS^•+^ and DPPH^•^ assays, respectively, performed for the leaves extracts, the mixture of leaves extracts and AgNPs, and purified AgNPs. The analyses were performed in triplicate (*n* = 3). TEAC-Trolox equivalent antioxidant capacity; GAE-gallic acid equivalent.

Scheme	Sample Name	ABTS^•+^ Assay		DPPH^•^ Assay	
		Inhibition %	mmol TEAC/L	Inhibition %	mg GAE/L
Plant extracts	CsDi	66.293 ± 0.019 ^a^	0.232 ± 0.001	15.213 ± 0.058 ^a^	9.426 ± 0.031
	CsDe	80.076 ± 0.011 ^b^	0.611 ± 0.001	16.808 ± 0.052 ^b^	10.282 ± 0.028
	CsSm	84.322 ± 0.139 ^c^	0.727 ± 0.004	11.820 ± 0.039 ^c^	7.606 ± 0.021
	CsSf	81.973 ± 0.014 ^d^	0.663 ± 0.001	29.923 ± 0.002 ^d^	17.318 ± 0.001
Extracts + AgNPs	PDi	86.065 ± 0.026 ^a^	0.775 ± 0.001	3.398 ± 0.065 ^a^	3.087 ± 0.035
	PDe	88.523 ± 0.037 ^b^	0.843 ± 0.001	1.560 ± 0.035 ^b^	2.101 ± 0.019
	PSm	89.185 ± 0.039 ^c^	0.861 ± 0.001	1.800 ± 0.007 ^c^	2.230 ± 0.004
	PSf	90.334 ± 0.047 ^d^	0.893 ± 0.001	2.323 ± 0.067 ^d^	2.510 ± 0.036
AgNPs	NPDi	88.772 ± 0.044 ^a^	0.850 ± 0.001	5.604 ± 0.009 ^a^	4.271 ± 0.005
	NPDe	89.529 ± 0.017 ^b^	0.871 ± 0.001	6.651 ± 0.026 ^ab^	4.833 ± 0.014
	NPSm	88.228 ± 0.017 ^c^	0.835 ± 0.001	6.670 ± 0.012 ^ab^	4.843 ± 0.007
	NPSf	73.109 ± 0.025 ^d^	0.419 ± 0.001	6.383 ± 0.007 ^b^	4.689 ± 0.004

The results are presented as mean ± standard deviation and superscript different letters (^a–d^) denote significant differences between the results on the same column with data for each sample type, after Tukey’s test for *p* < 0.05. Means with superscripts having the same letter in the column with data for each sample type are not significantly different.

**Table 4:** The main recorded FT-IR ATR bands on 4000- 600 cm^−1^ range.

FT-IR Range(cm^−1^)	Main FT-IR Bands (cm^−1^)							
	CsDi	CsDe	CsSf	CsSm	PDi	PDe	PSf	PSm
3550–3100	3290.91	3296.60	3280.63	3286.07	3326.57	3262.02	3253.32	3252.98
3100–2850	2924.22	2926.59	2925.92	2925.24	2925.64	2931.89	2929.99	2930.31
	2888.83	2898.46	2897.24	2899.24	2884.24	2899.38	2898.82	2898.72
	2859.24	2864.85	2859.74	2856.32	2858.99	2861.87	2858.78	2859.16
1700–1500	1637.94	1637.02	1639.24	1638.92	1636.24	1635.92	1638.26	1637.13
1500–1200	1404.76	1407.54	1405.00	1405.01	-	-	-	-
	-	-	-	-	1368.33	1368.57	1368.01	1368.52
1200–900	1043.20	1043.82	1043.13	1043.80	1043.19	1043.91	1043.20	1043.68
900–600	922.38	923.88	922.37	924.14	922.12	924.21	923.27	923.73
	866.29	870.02	872.20	870.01	866.16	870.96	872.05	870.77
	778.69	822.41	825.41	825.80	824.24	824.71	824.44	824.82
	778.69	777.53	779.38	780.17	779.11	779.87	781.58	781.07
	712.03	712.32	712.01	713.15	711.83	711.47	9712.21	711.55
	672.59	669.25	669.25	670.55	672.28	668.35	667.45	675.57
	616.24	626.27	621.84	618.38	617.04	628.33	621.54	612.38

**Table 5:** DLS data recorded for *Cannabis sativa* leaf extracts with AgNPs and purified AgNPs.

Sample Type	Sample Name	Z-Ave [nm]	PdI	Peak 1: d [nm] (Weight (%))	Peak 2: d [nm] (Weight (%))	Peak 3: d [nm] (Weight (%))
Extracts + AgNPs	PDi	79.8	0.371	104.3 (71%)	33.9 (17%)	3416 (12%)
	PDe	139.9	0.424	266.0 (62%)	74.2 (38%)	0
	PSm	119.2	0.367	198.8 (62%)	75.5 (38%)	0
	PSf	102.1	0.376	180.8 (55%)	75.1 (45%)	0
AgNPs	NPDi	147.1	0.569	258.6 (66%)	74.5 (31%)	5337 (3%)
	NPDe	113.7	0.338	156.8 (95%)	32.1 (5%)	0
	NPSm	195.1	0.478	133.7 (59%)	872.5 (41%)	0
	NPSf	104.3	0.293	77.2 (52%)	210.4 (48%)	0

**Table 6:** Inhibition zones of *Cannabis sativa* leaf extracts without and with AgNPs. CsDi—Diana leaves extracts; PDi—Diana leaves extracts + AgNPs; CsDe—Denise leaves extracts; PDe—Denise leaves extracts + AgNPs; CsSm—Silvana male leaves extract; PSm—Silvana male leaves extract + AgNPs; CsSf—Silvana female leaves extract; PSf—Silvana female leaves extract + AgNPs.

Inhibition Zones Diameter (mm)										
Strain Name	CsDi	CsDe	CsSf	CsSm	PDi	PDe	PSf	PSm	Amikacin	Gentamicin
*K. pneumoniae*	6	7 ± 0.1	7 ± 0.17	8 ± 0.1	12 ± 0.17	14 ± 0.17	10 ± 0.0	12 ± 0.26	14 ± 0.2	ND
*P. fluorescens*	6	6	6	6	11 ± 0.2	10 ± 0.2	11 ± 0.1	13 ± 0.17	ND	20 ± 0.1
*E. coli*	7 ± 0.1	7 ± 0.1	7 ± 0.2	8 ± 0.1	10 ± 0.1	11 ± 0.26	12 ± 0.26	10 ± 0.1	20 ± 0.17	ND
*S. aureus*	7 ± 0.1	7 ± 0.1	7 ± 0.1	7 ± 0.1	12 ± 0.17	13 ± 0.1	13 ± 0.0	13 ± 0.1	ND	21 ± 0.1

ND—not determined; for the values of 6 mm we considered no antibacterial activity as 6 mm is the diameter of the test discs.

## Supplementary Materials

The following are available online, Figure S1. FT-IR ATR spectra of the *Cannabis sativa* leaves extracts with blue [CsDi (a), CsDe (b), CsSf (c), CsSm (d)) and of mixtures of extracts+biosynthesized silver nanoparticles AgNPs with red: PDi (a) PDe (b), PSf (c), PSm (d)]. Figure S2. Images with inhibition zones against bacteria: *Escherichia coli* (a-1) PDe, CsDe, Amikacin (positive control), negative control (water: H_2_O); (a-2) PDi, CsDi, Amikacin (positive control), negative control (water); (b-1) PSf, CsSf, Amikacin, negative control (water); (b-2) PSm, CsSm, Amikacin (positive control), control. *Pseudomonas fluorescens*: (c-1) PDi, CsDi, Gentamicin (positive control), negative control (water); (c-2) PDe, CsDe, Gentamicin (positive control), negative control (water); (d-1) PSm, CsSm, Gentamicin (positive control), negative control (water); (d-2) PSf, CsSf, Gentamicin (positive control), negative control (water); *Klebsiella pneumoniae*: (e-1) PSm, CsSm, Amikacin (positive control), negative control (water); (e-2) PDe, CsDe, Amikacin (positive control), negative control (water); (f-1) PDi, CsDi, Amikacin(positive control), negative control (water); and (f-2) PSf, CsSf, Amikacin (positive control), negative control (water); Staphyloccocus aureus: (g-1) PDi, CsDi, Amikacin (positive control), negative control (water); and (g-2) PDe, CsDe, Amikacin (positive control), negative control (water); Antibiotic is abbreviated as Atb. (a’–f’ are the reversed Petri dishes).

## Sample Availability

Samples of the compounds are not available from the authors.
