# Supplementary material for: Green Synthesis, Characterization, and Antibacterial Properties of Silver Nanoparticles Obtained by Using Diverse Varieties of Cannabis sativa Leaf Extracts
Source: Molecules. 2021 Jul 1;26(13):4041. doi: 10.3390/molecules26134041 (PMC8271394; doi:10.3390/molecules26134041)
Supplement: Supplementary file 1 [file molecules-26-04041-s001.zip › molecules-1258036-supplementary.pdf]

Green synthesis, characterization, and antibacterial properties of silver nanoparticles obtained by using diverse varieties of *Cannabis sativa* leaf extracts

Supplementary Information

Csakvari, A.C.; Moisa, C.; Radu , D.G.; Olariu, L.M.; Lupitu, A.I.; Panda, A.O.; Pop, G.; Chambre, D.; Socoliuc, V.; Copolovici, L.; Copolovici, D.M.

FT-IR ATR spectra of the samples are presented in Figure S1.

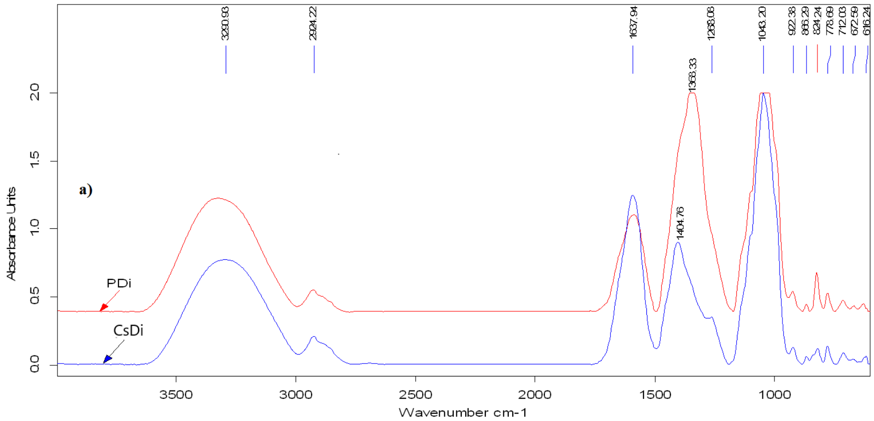

(a)

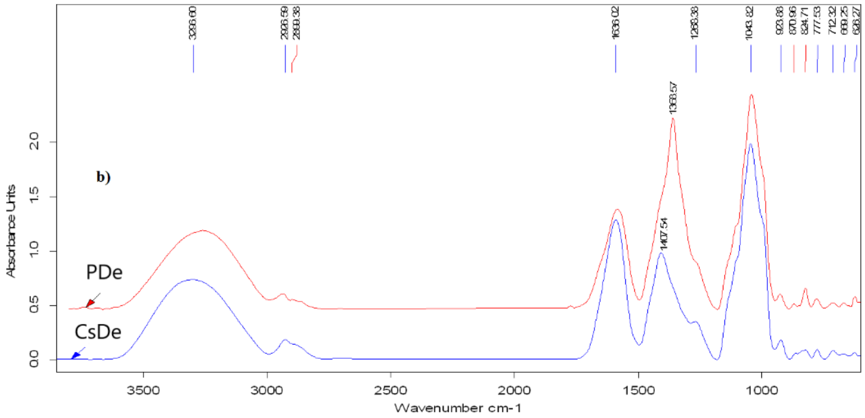

(b)

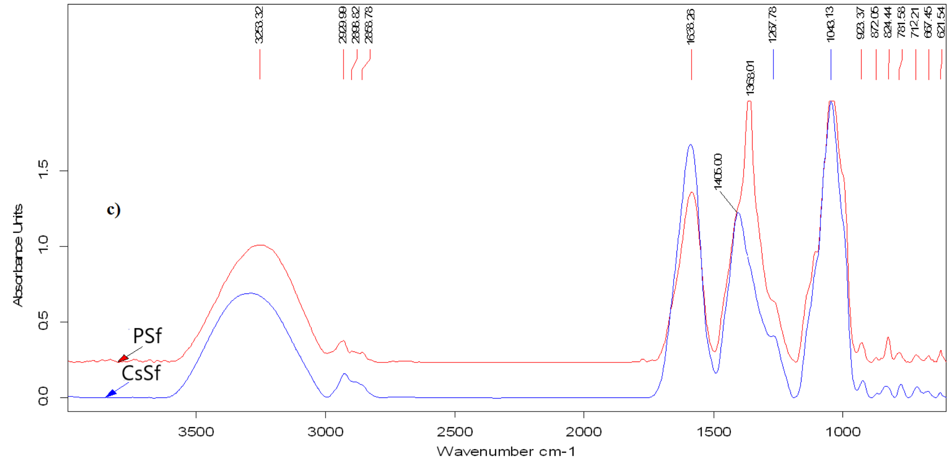

(**c**)

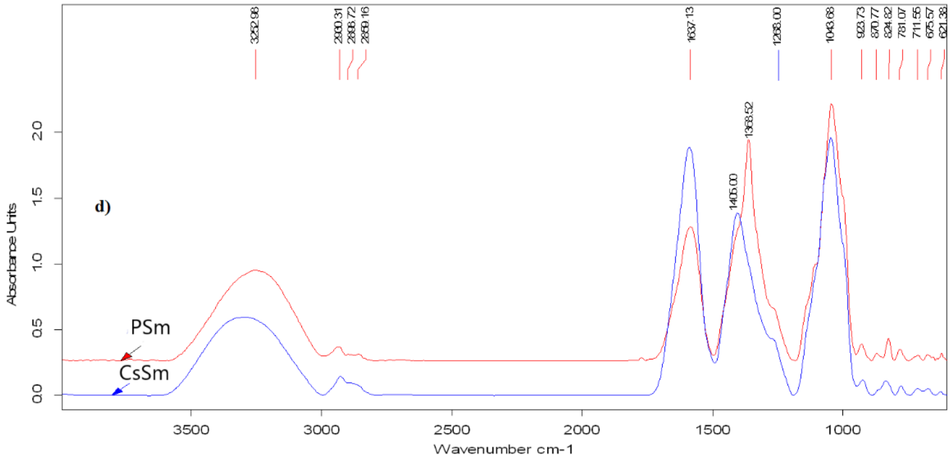

(**d**)

Figure S1. FT-IR ATR spectra of the *Cannabis sativa* leaves extracts with blue [CsDi (a), CsDe (b), CsSf (c), CsSm (d)) and of mixtures of extracts+biosynthesized silver nanoparticles AgNPs with red: PDi (a) PDe (b), PSf (c), PSm (d)].

The antibacterial activity of the leaves *Cannabis sativa* extracts without or with biosynthesized AgNPs was evaluated on four bacterial strains, namely 3 Gram-negative bacteria: *Escherichia coli*, *Klebsiella pneumoniae, Pseudomonas fluorescens*, and one Gram-positive bacteria *Staphyloccocus aureus* using a disc agar diffusion method (Figure S2).

(**a**) 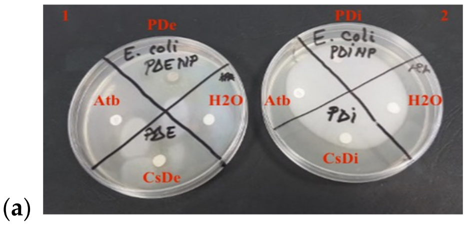

(**a′**) 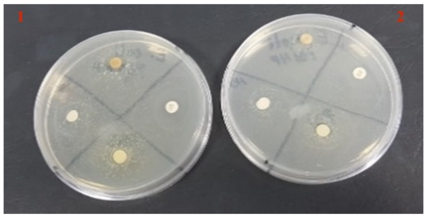

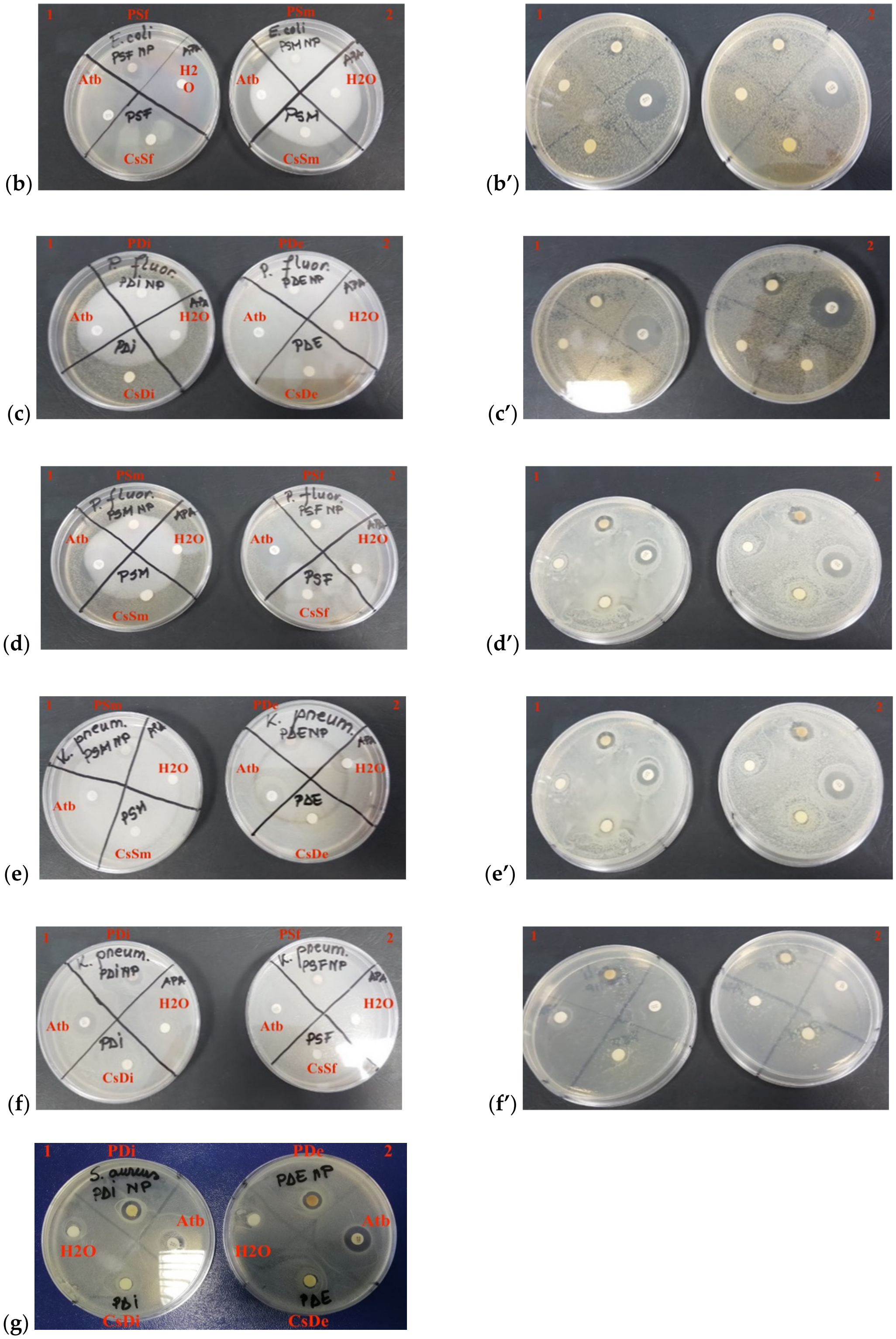

Figure S2. Images with inhibition zones against bacteria: *Escherichia coli* (a-1) PDe, CsDe, Amikacin (positive control), negative control (water: H_2O); (a-2) PDi, CsDi, Amikacin (positive control), negative control (water); (b-1) PSf, CsSf, Amikacin, negative control (water); (b-2) PSm, CsSm, Amikacin (positive control), control. *Pseudomonas fluorescens*: (c-1) PDi, CsDi, Gentamicin (positive control), negative control (water); (c-2) PDe, CsDe, Gentamicin (positive

control), negative control (water); (d-1) PSm, CsSm, Gentamicin (positive control), negative control (water); (d-2) PSf, CsSf, Gentamicin (positive control), negative control (water); *Klebsiella pneumoniae*: (e-1) PSm, CsSm, Amikacin (positive control), negative control (water); (e-2) PDe, CsDe, Amikacin (positive control), negative control (water); (f-1) PDi, CsDi, Amikacin(positive control), negative control (water); and (f-2) PSf, CsSf, Amikacin (positive control), negative control (water); *Staphyloccocus aureus:* (g-1) PDi, CsDi, Amikacin (positive control), negative control (water); and (g-2) PDe, CsDe, Amikacin (positive control), negative control (water); Antibiotic is abbreviated as Atb. (a' – f' are the reversed Petri dishes).
